# Colchicine for Prevention of Post-Cardiac Surgery and Post-Pulmonary Vein Isolation Atrial Fibrillation: A Meta-Analysis

**DOI:** 10.31083/j.rcm2312387

**Published:** 2022-11-28

**Authors:** Xuesi Wang, Xiaodong Peng, Yukun Li, Rong Lin, Xinmeng Liu, Yanfei Ruan, Changsheng Ma, Nian Liu

**Affiliations:** ^1^Department of Cardiology, Beijing Anzhen Hospital, Capital Medical University, 100029 Beijing, China; ^2^National Clinical Research Center for Cardiovascular Diseases, 100029 Beijing, China

**Keywords:** colchicine, atrial fibrillation, post-cardiac surgery atrial fibrillation, post-pulmonary vein isolation

## Abstract

**Background:**

Post-cardiac procedure atrial fibrillation (PCP-AF) is a significant medical problem. Inflammation is one of the key factors in the pathogenesis of PCP-AF. As a classical anti-inflammatory drug, colchicine may prevent the occurrence of PCP-AF. This meta-analysis of 12 randomized controlled trials (RCTs) analyzed the feasibility and safety of colchicine for the prevention of PCP-AF.

**Methods:**

PubMed, EMBASE, Web of Science, the Cochrane Library, and Google Scholar were retrieved for RCTs on the efficacy of colchicine in preventing atrial fibrillation. The primary endpoint was the diagnosis of PCP-AF, which includes cardiac surgery or pulmonary vein isolation. Evaluation was performed with estimated odds ratios (OR) and 95% confidence intervals (CI).

**Results:**

In this meta-analysis, 12 RCTs were selected and a total of 2297 patients were included. Colchicine therapy was associated with a reduced incidence of PCP-AF both in post-cardiac surgery (OR: 0.62; 95% CI: 0.49–0.78, *p *< 0.0001, $I^{2}$ = 0%), and in post-pulmonary vein isolation (OR: 0.43; 95% CI: 0.30–0.62, *p *< 0.0001, $I^{2}$ = 0%). Colchicine therapy was associated with increased side effects (OR: 2.81; 95% CI: 1.96–4.03, *p *< 0.00001, $I^{2}$ = 26%).

**Conclusion:**

Colchicine can effectively prevent post-cardiac operative atrial fibrillation and relapse of atrial fibrillation after pulmonary vein isolation (PVI). However, colchicine can also increase the incidence of side effects, mainly gastrointestinal adverse events. More studies are needed to find a more appropriate treatment dose and time.

## 1. Introduction

Atrial fibrillation is one of the most common arrhythmias in the clinic and has become a cardiovascular epidemic in the 21st century [1]. Atrial Fibrillation is related to cardiac procedures, including coronary artery bypass graft, valve surgery, aortic surgery, and pulmonary vein isolation [2]. PCP-AF will increase the length of hospital stay, mortality, and economic burden, so the prevention and treatment of post-operative atrial fibrillation are critical [3, 4]. The pathogenesis of atrial fibrillation is complex, and inflammation plays an important role. Inflammation after cardiac operation and radiofrequency ablation is closely related to PCP-AF [5]. As a classic anti-inflammatory drug, colchicine may be a potential drug for the prevention and treatment of PCP-AF. Previous studies have demonstrated the preventive effect of colchicine on PCP-AF [6, 7]. However, other studies have shown that colchicine doesn’t significantly reduce the incidence of PCP-AF, and colchicine is related to more adverse reactions [8, 9]. Therefore, the benefit of colchicine to post-operative patients cannot be determined. This study analyzed the feasibility and safety of colchicine in preventing PCP-AF by integrating relevant data from various RCTs.

## 2. Methods

### 2.1 Search Strategy

A study was planned and performed using methods specified in the Preferred Reporting Items for Systematic Reviews and Meta-Analyses (PRISMA) guidelines [10].

We systematically searched PubMed, EMBASE, Web of Science, the Cochrane Library, and Google Scholar using the following keywords: atrial fibrillation, atrial, or fibrillation, and colchicine. Literature searches were completed on March 23, 2022. The list of references in selected articles was also searched to find a study that met the inclusion criteria. No language or study type restriction was used for the initial extraction of the data. Retrieval also does not restrict subtitles. All citations and related literatures were also reviewed. All non-English manuscripts were considered for inclusion in the meta-analysis after translation.

### 2.2 Study Selection

Trials were eligible if they met the following criteria: (1) randomized controlled trials; (2) head-to-head comparison between colchicine versus placebo; (3) participants included in the study underwent cardiac surgery and/or atrial fibrillation radiofrequency ablation. PCP-AF was defined as clinically significant AF or documented episode of AF lasting at least 30 s following any cardiac surgery or PVI [11]. The primary endpoint was the diagnosis of PCP-AF.

### 2.3 Data Extraction

**Fig. 1.**
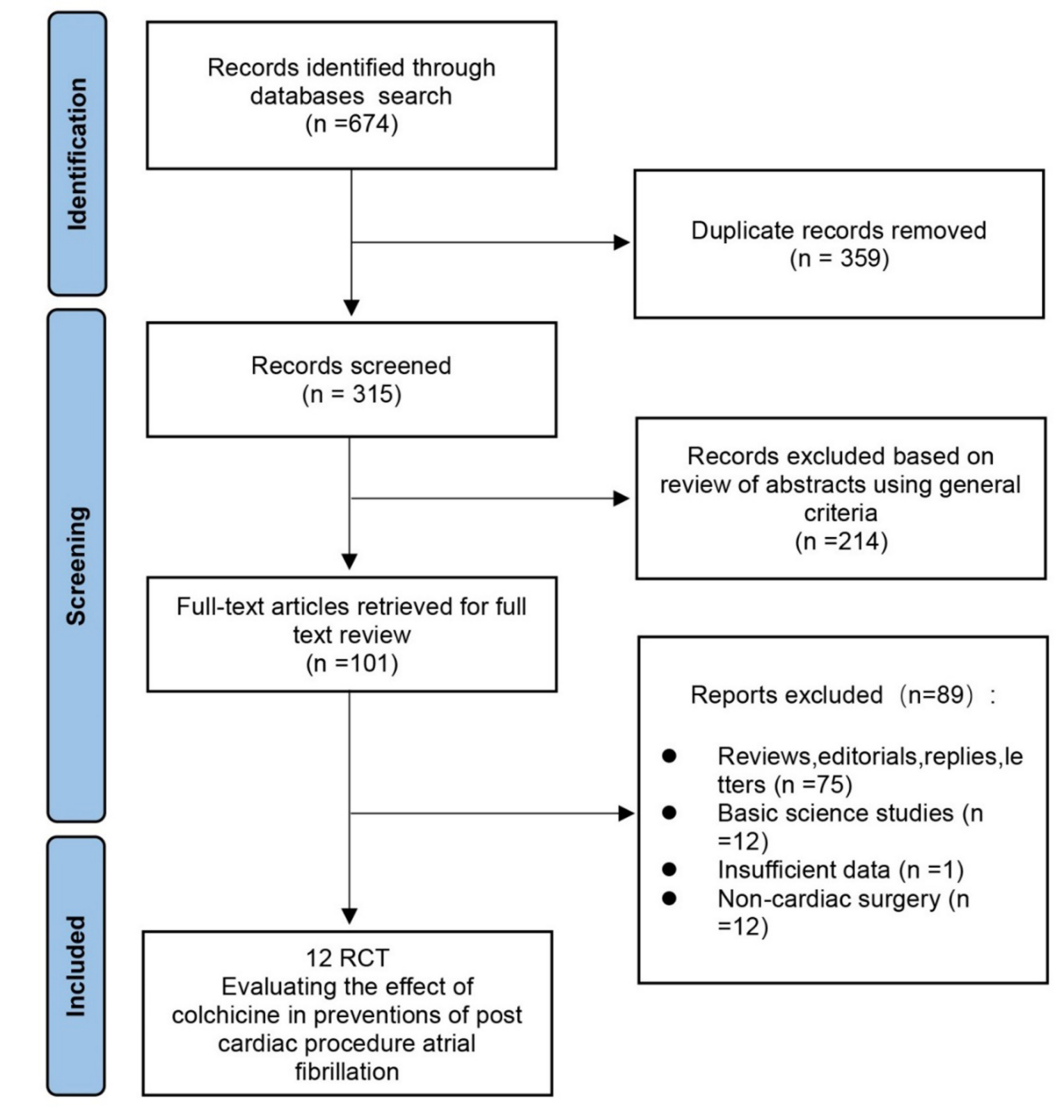
**Summary of the selection process of randomized controlled trials included in the meta-analysis**.

Two reviewers (W.X.S. and L.Y.K.) independently screened all identified titles or abstracts, and full-text was reviewed for studies that satisfied the inclusion criteria. A total of 14 articles were identified after the independent searches by two reviewers. Then after discussion, incomplete data and non-cardiac surgery were excluded, and 12 RCTs were included (Fig. 1) [6, 7, 8, 9, 12, 13, 14, 15, 16, 17, 18, 19]. We extracted study characteristics such as study design, baseline characteristics, sample size, type of procedures, intervention, primary and secondary outcomes, follow-up duration and side events.

### 2.4 Risk of Bias

Cochrane’s risk of bias tool has been utilized to assess each study’s risk of bias.

### 2.5 Statistical Analysis

RCTs were included in the study or trial-level pooled analysis to evaluate the feasibility and safety of colchicine for the prevention of PCP-AF.

Cochrane Review Manager (RevMan) 5.4.1 (Cochrane Collaboration, Haymarket, London, UK) was used for meta-analysis. Heterogeneity was evaluated using chi-square, Tau-square, and I-square ($I^{2}$) statistics. The pooled odds ratio (ORs) estimates with 95% confidence intervals (CIs) were conducted using a random-effects model and the Mantel-Haenszel method. We regarded $I^{2}$ of <25%, 25% to 50%, and >50% as low, moderate, and high heterogeneity amounts, respectively. Publication bias was assessed with funnel plot. Subgroup analyses were performed on the type of cardiac procedure. Pearson chi-square test was performed using SPSS 24 (IBM, Armonk, NY, USA) to compare the categorical variables of patients with colchicine and placebo. Age differences between patients in the colchicine and placebo groups were compared using an independent-sample *T* tests. Sensitivity analysis was performed by repeating the analysis five times and removing one study at a time. *p*-values < 0.05 was considered statistically significant.

## 3. Results

### 3.1 Study Characteristics

Our meta-analysis included 12 RCT studies. Eight RCTs investigated the effect of colchicine on preventing atrial fibrillation in post-cardiac patients, and 4 RCTs explored the effect of colchicine in preventing the recurrence of atrial fibrillation after pulmonary vein isolation. A total of 2297 patients were included in this study, with 1123 patients randomized to receive colchicine and 1174 patients to receive placebo. All study characteristics and baseline data can be viewed in Table 1 (Ref. [6, 7, 8, 9, 12, 13, 14, 15, 16, 17, 18, 19]) and Table 2, respectively. Eight RCTs were prospective, double-blinded, randomized, placebo-controlled trials. Two other studies were not double-blind, and two studies did not report research methods. Patients underwent cardiac surgery including coronary artery bypass grafting (CABG), aortic surgery, valvular surgery, or combined. Most patients were treated with a 1.0–2.0 mg loading dose of colchicine before or after the procedure, followed by a maintenance dosage of 0.5–1.0 mg/day. As for the intervention time of colchicine, there are significant differences among the groups, the shortest one is only 5 days, and the longest one is 3 months. The follow-up duration ranged from 7 days to one year. All post-procedure atrial fibrillation was measured by a 12-lead electrocardiogram (ECG) or continuous cardiac monitoring. There were no significant differences in baseline characteristics between the colchicine group and the placebo group.

**Table 1.** **Characteristics of Included Studies**.

Study name	Sample size	Study design	Type of procedures	Intervention	AF monitoring method	Follow up duration	Primary end point
Imazio *et al*. 2010 [6]	336	Randomized, double-blind, placebo controlled	CABG, aortic surgery, valvular surgery, combined	Colchicine 1 mg bid, day 3 PO, then 0.5 mg bid for 1 month	Continuous ECG monitoring, 12-lead ECG recordings	1 month	AF
Deftereos *et al*. 2012 [12]	161	Randomized, double-blind, placebo-controlled	Pulmonary vein isolation	Colchicine 0.5 mg bid for 3 months	Holter	3 months	AF recurrence,Episodes of atrial flutter
Egami *et al*. 2013 [14]	62	Not reported	Pulmonary vein isolation	Colchicine 0.5 mg qd for 2 weeks	Not reported	2 weeks	AF recurrence
Deftereos *et al*. 2014 [13]	206	Randomized, double-blind, placebo-controlled	Pulmonary vein isolation	Colchicine 0.5 mg bid for 3 months	Holter	3 months and 12 months	AF recurrence,Episodes of atrial flutter
Imazio *et al*. 2014 [8]	360	Randomized, double-blind, placebo controlled	CABG, aortic surgery, valvular surgery, combined	Colchicine 0.5 mg bid, 48–72 h before surgery and continued for 1 month	Continuous ECG monitoring ≥5 days post-surgery, 12 lead ECG daily	3 months	AF
Sarzaeem *et al*. 2014 [7]	216	Randomized, double-blind, placebo controlled	CABG	Colchicine 1 mg the night before and on the morning of surgery, then 0.5 mg bid for 5 days	Not reported	6 months	AF
Egami *et al*. 2015 [15]	122	Not reported	Pulmonary vein isolation	Colchicine 0.5 mg/d for 2 weeks	Not reported	3 months	AF recurrence
Zarpelon *et al*. 2015 [16]	140	Randomized, open-label	Myocardial revascularization surgery	Colchicine 1 mg bid, in the preoperative period, followed by 0.5 mg bid until hospital discharge	Continuous cardiac monitoring and 12-lead electrocardiogram (ECG)	Until discharge	AF
Tabbalat *et al*. 2016 [9]	360	Randomized, open-label	Open-heart surgeries	Colchicine 2 mg 12–24 hours prior to surgery and 1 mg 4 hours before or immediately after surgery, then 0.5 mg bid until hospital discharge	Continuous cardiac monitoring and 12-lead electrocardiogram (ECG)	Until discharge	AF
Mashayekhi *et al*. 2020 [17]	81	Randomized, double-blind, placebo controlled	Open-heart surgeries	Colchicine 1 mg bid for first day after surgery, and then received 1 mg qd for one month	ECG	Not reported	Post-pericardiotomy syndrome
Tabbalat *et al*. 2020 [18]	152	Randomized, double-blind, placebo controlled	Open-heart surgeries	1 mg of colchicine 12 to 24 hours prior to surgery, colchicine 0.5 mg immediately after their surgery and 0.5 mg qd until hospital discharge	ECG	Until discharge	AF
Shvartz *et al*. 2022 [19]	101	Randomized, double-blind, placebo controlled	CABG and/or AVR	1 mg of colchicine 24 h before the surgery , as well as on days 2–5 in the postoperative period	ECG	7 days	AF

ECG, electrocardiogram; CABG, coronary artery bypass grafting; AF, atrial fibrillation; AVR, aortic valve replacement.

**Table 2.** **Baseline characteristics**.

	Colchicine (n = 1123)	Placebo (n = 1174)	*p* value
Age	64.3 ± 10.6	63.3 ± 11.0	0.48
Male	662/941	686/956	0.50
Hypertension	604/941	610/956	0.86
Diabetes	319/941	290/956	0.10
CHF	116/739	121/765	0.95
COPD	32/397	29/300	0.46
Smoking	216/774	225/787	0.77
PVI	256/1123	295/1174	0.39
Valvular surgery	158/605	149/633	0.29
CABG surgery	332/605	368/633	0.25
Aortic surgery	22/605	18/633	0.43
Combined surgery	94/605	97/633	0.92

COPD, chronic obstructive pulmonary disease; CHF, congestive heart failure; PVI, pulmonary vein isolation; CABG, coronary artery bypass grafting; AF, atrial fibrillation. Values are n/N (%) or mean ± SD.

### 3.2 Quality Assessment

Sensitivity analysis showed that the overall conclusion was not affected after excluding individual studies (details omitted). **Supplementary Fig. 1** shows the quality assessment of the included studies.

### 3.3 Prevention of Post-Cardiac Procedure Atrial Fibrillation

**Fig. 2.**
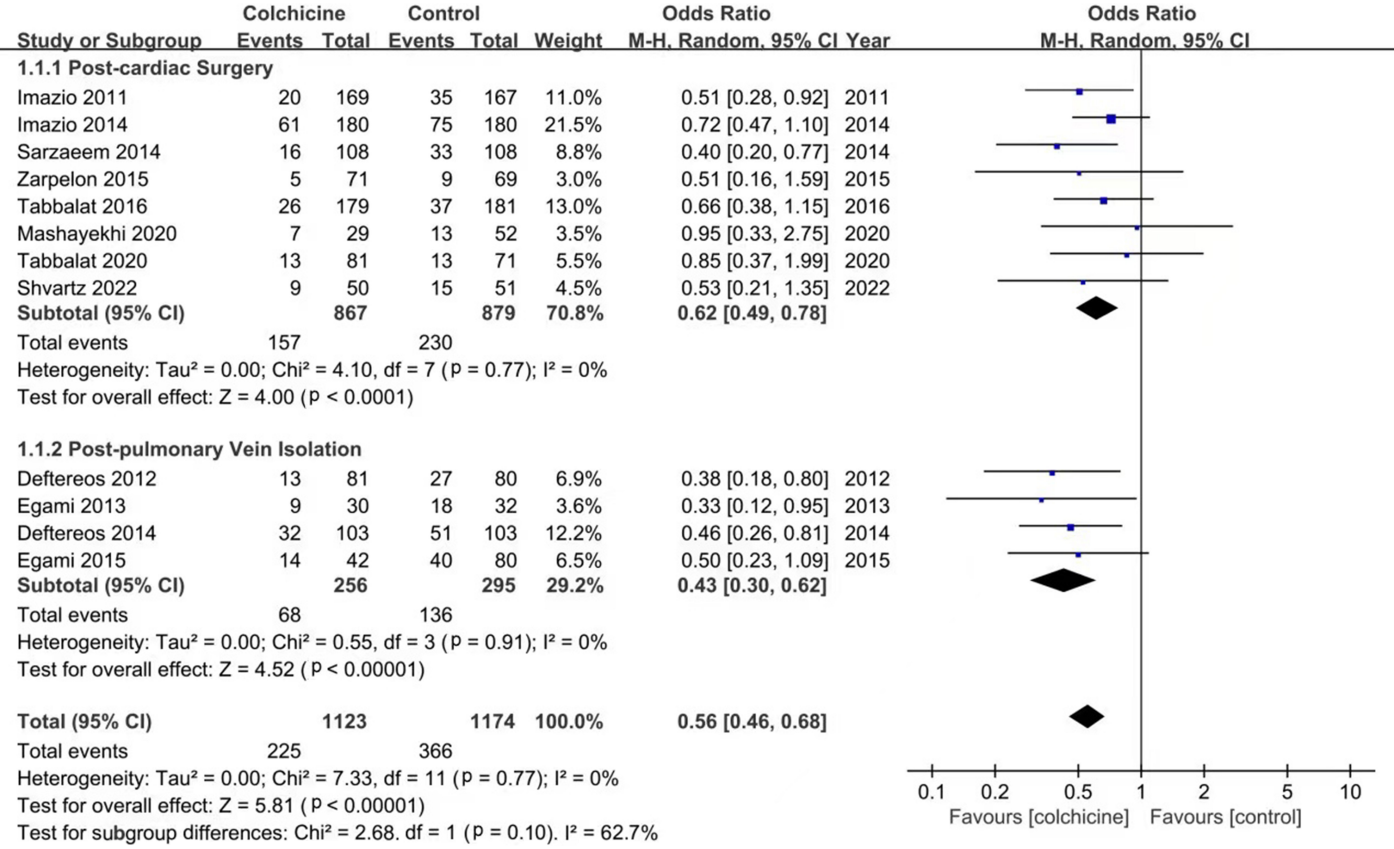
**Forest plot showing estimated odds ratios of PCP-AF with colchicine use versus placebo**.

According to the meta-analysis of 12 studies, the use of colchicine can significantly reduce the incidence of PCP-AF (OR: 0.56; 95% CI: 0.46–0.68, *p *< 0.00001, $I^{2}$ = 0%) (Fig. 2). 20% (225/1123) who received colchicine experienced PCP-AF versus 31% (366/1174) control patients. The heterogeneity among the studies calculated using the random method was low ($I^{2}$ = 0%, Chi-square = 7.33, df = 11, *p *< 0.00001).

In addition, subgroup analysis showed that the incidence of PCP-AF was statistically significantly reduced in the colchicine group after cardiac surgery (OR: 0.62; 95% CI: 0.49–0.78, *p *< 0.0001, $I^{2}$ = 0%) and after post-pulmonary vein isolation (OR: 0.43; 95% CI: 0.30–0.62, *p *< 0.0001, $I^{2}$ = 0%) (Fig. 2). Atrial fibrillation occurred in 18% (157/867) post-cardiac patients who received colchicine, compared with 26% (230/879) patients who did not. However, the sample size of studies on post-pulmonary vein isolation is relatively small; 26% (68/256) of patients in the colchicine group had a recurrence of atrial fibrillation, while 46% (136/295) in the control group.

**Fig. 3.**
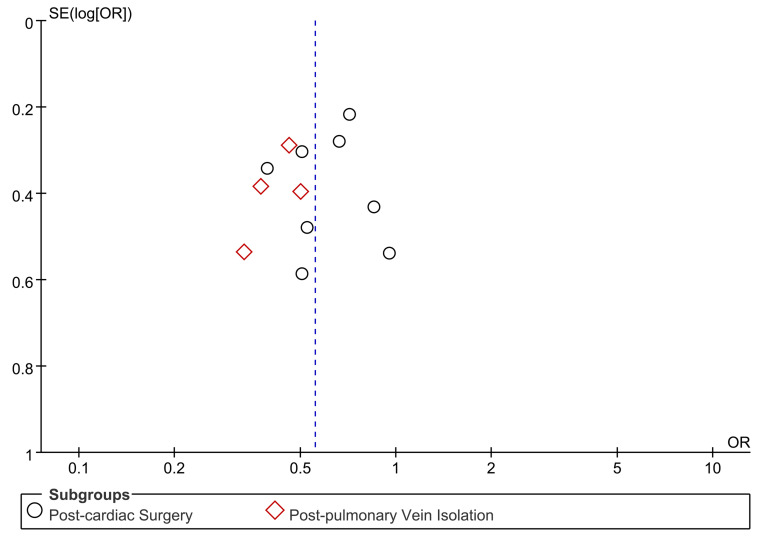
**Funnel plot of standard error by estimated odds ratio**.

The funnel plot indicates possible publication bias. The studies were evenly distributed on the plot around the summary effect size (Fig. 3). This proves that the publication bias of this study is slight.

### 3.4 Adverse Events

**Fig. 4.**
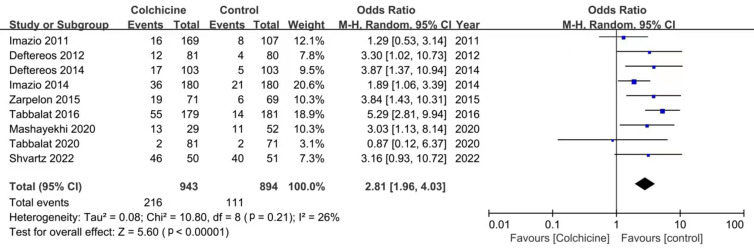
**Forest plot showing overall adverse events**.

**Fig. 5.**
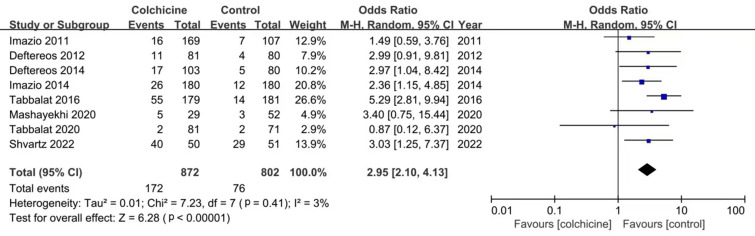
**Forest plot showing gastrointestinal side effects**.

Adverse events reported in various studies include nausea, lack of appetite, diarrhea, anorexia, and other gastrointestinal adverse reactions, abdominal pain, hepatotoxicity, myotoxicity, bone-marrow toxicity, alopecia, and anorexia. In general, the meta-analysis showed that the incidence of side events was higher in the colchicine group (OR: 2.81; 95% CI: 1.96–4.03, *p *< 0.00001, $I^{2}$ = 26%) (Fig. 4). Gastrointestinal side effects were the commonest adverse effects. The incidence of gastrointestinal side events in colchicine group was significantly higher than that in placebo group (OR: 2.95; 95% CI: 2.10–4.13, *p *< 0.00001, $I^{2}$ = 3%) (Fig. 5).

## 4. Discussion

This meta-analysis based on 12 RCTs showed that perioperative use of colchicine significantly reduced the incidence of PCP-AF. Our results are consistent with previous meta-analyses and confirm the effect of colchicine on PCP-AF [20, 21, 22]. This study is the largest meta-analysis to date, involving 2297 patients, of whom 1746 underwent cardiac surgery, and 551 underwent pulmonary vein isolation. Subgroup analysis of cardiac surgery and radiofrequency ablation also suggests that colchicine effectively prevents atrial fibrillation.

Post-operative atrial fibrillation (POAF) is defined as new-onset atrial fibrillation after surgery or intervention [23]. The prevalence of POAF is almost between 20% and 40% [24, 25]. The incidence of POAF after thoracic surgery is lower than that after cardiac surgery [26, 27]. In view of the close relationship between cardiac operation and post-operative atrial fibrillation and the high recurrence rate of atrial fibrillation after pulmonary vein isolation [28], our study focused on the role of colchicine in the prevention of PCP-AF. Like other types of atrial fibrillation, PCP-AF is caused by ectopic firing and/or re-entry. The vulnerable atrial substrate resulting from atrial structure remodeling, connexin remodeling, electrical remodeling, and ${\mathrm{Ca}}^{2+}$-handling remodeling triggered activity and maintains re-entry. This process is the result of the joint action of many factors. After the cardiac procedure, the increase in blood norepinephrine concentration, sympathetic tension, and inflammatory process all play an essential role in the occurrence and development of atrial fibrillation [29, 30, 31]. The inflammatory process may be the common terminal pathway of cardiac structural remodeling and electrical remodeling by various mechanisms. Several studies have shown that IL-2, IL-6, and CRP levels are related to PCP-AF occurrence [32, 33, 34], and corticosteroids can reduce the incidence of PCP-AF after cardiac surgery or radiofrequency catheter ablation [35, 36]. Meanwhile, the level of nuclear factor-κB (NF-κB) in the atrial tissues of POAF patients is increased, and the higher level of NF-ϵB protein initiates and triggers the activation of NLRP3 inflammasome, which promotes the progression of inflammation [37, 38].

Previous studies have found that short-term post-operative corticosteroids can reduce the incidence of PCP-AF, which confirms the feasibility of anti-inflammatory drugs to prevent PCP-AF [35, 36]. Colchicine is a cheap and commonly used anti-inflammatory drug. It inhibits the activity of neutrophils and reduces the adhesion between inflammatory cells and endothelium by inhibiting tubulin polymerization, destroying the cytoskeleton, inhibiting division and intracellular transport, and finally plays an anti-inflammatory role [39, 40, 41, 42]. Imazio *et al*.’s [6] COPPS test first proved colchicine’s preventive effect on atrial fibrillation after cardiac surgery in 2011. Subsequently, another RCT study published by Sarzaeem *et al*. [7] in 2014 further supports the preventive effect of colchicine on POAF. However, although all the other six studies, including the COPPS2 study, concluded that the incidence of atrial fibrillation in the colchicine group was low, they failed to produce a statistical difference from the control group [6, 7, 8, 9, 16, 17, 18, 19]. Through our meta-analysis of all studies, we concluded that colchicine can effectively prevent post-operative atrial fibrillation. The difference between a single study and a meta-analysis may be due to the small number of samples of a single RCT study, which cannot well represent the whole from the part. The research heterogeneity in meta-analysis is small, and the combined sample size is expanded, reflecting the clinical significance better. There are 4 RCT studies after radiofrequency ablation, and most of them have proved that colchicine can prevent the recurrence of atrial fibrillation after pulmonary vein isolation, except for the study of Egami *et al*. [12, 13, 14, 15]. The specific mechanism of colchicine in preventing PCP AF is not precise. In addition to its anti-inflammatory effect, colchicine can inhibit microtubule polymerization and regulate the phosphorylation of calcium channels, thus affecting intracellular calcium homeostasis and reducing the possibility of calcium overload-induced tachyarrhythmia. *In vitro* studies have shown that colchicine can shorten the duration of collagen-induced action potential in HL-1 cells [43, 44]. On the other hand, the process of microtubule assembly leads to increased secretion of extracellular matrix (ECM) such as type I collagen [45], and higher contents of ECM increase the occurrence of atrial fibrillation through structural and electrical remodeling [46, 47]. Therefore, colchicine may reduce myocardial remodeling and prevent atrial fibrillation by reducing ECM accumulation.

Because of increased hospital stay, mortality, and hospitalization burden caused by PCP-AF, colchicine should be a suitable secondary preventive drug [3, 4]. However, the side effects of colchicine limit its large-scale application. Our results emphasize that the incidence of total side effects and gastrointestinal side events in the colchicine group is higher than that in the control group, which suggests that colchicine should be used in patients with high-risk factors of atrial fibrillation, such as advanced age, obesity, family history of atrial fibrillation, long-term smoking and drinking history, heart failure, diabetes, valvular disease and chronic obstructive pulmonary disease [48]. A weight-adjusted dose (0.5 mg maximum for patients less than 70 kg and 0.5 mg twice daily for patients ≥70 kg) and a seizure avoidance dose may help decrease gastrointestinal side events and maintain the same therapeutic effect as in the previous trial without load. Careful consideration of colchicine drug interactions and side events and using adjusted doses for weight and creatinine clearance may help decrease the incidence of adverse effects and improve compliance and treatment outcomes.

## 5. Limitation

First, the surgical methods in the studies we included are heterogeneous. Most of the research inclusion criteria are different kind of cardiac surgery. Shvartz *et al*. [19] included patients with coronary artery bypass grafting and aortic valve replacement, while Sarzaeem *et al*. [7] and Zarpelon *et al*. [16] only included patients with coronary artery bypass grafting. Other sources of heterogeneity include different dosages and times of colchicine administration and different follow-up times.

On the other hand, the detection of atrial fibrillation is insufficient in all studies. Due to the failure to continuously monitor the ECG status of patients for a long time, most studies choose to check 12 lead ECG or Holter regularly or when there are symptoms to detect atrial fibrillation, but this will ignore some paroxysmal atrial fibrillation or asymptomatic atrial fibrillation. However, considering that the interference effect of undetected atrial fibrillation on the colchicine and control groups is the same, it may have little impact on our research results. The two studies of Egami *et al*. [7, 14, 15] were published in abstracts, and the full text was not obtained, so the data were not comprehensive.

## 6. Conclusions

Colchicine can effectively prevent post-cardiac operative atrial fibrillation and recurrence of atrial fibrillation after PVI. However, colchicine can also increase the incidence of side effects, mainly gastrointestinal side effects. In the future, more studies are needed to find a more appropriate treatment dose and time to balance the contradiction between treatment and side effects.

## Patient and Public Involvement

Patients and/or the public were not involved in the design, or conduct, or reporting, or dissemination plans of this research.

## Supplementary Material

Supplementary material associated with this article can be found, in the online version, at https://doi.org/10.31083/j.rcm2312387.
