# Supplementary material for: Colchicine for Prevention of Post-Cardiac Surgery and Post-Pulmonary Vein Isolation Atrial Fibrillation: A Meta-Analysis
Source: Rev Cardiovasc Med. 2022 Nov 28;23(12):387. doi: 10.31083/j.rcm2312387 (PMC11270460; doi:10.31083/j.rcm2312387)
Supplement: Supplementary file 1 [file 2153-8174-23-12-387-s1.zip › 2153-8174-23-12-387-s1/Supplementary File.docx]

Supplementary Fig. 1. Risk of bias assessments for included studies:


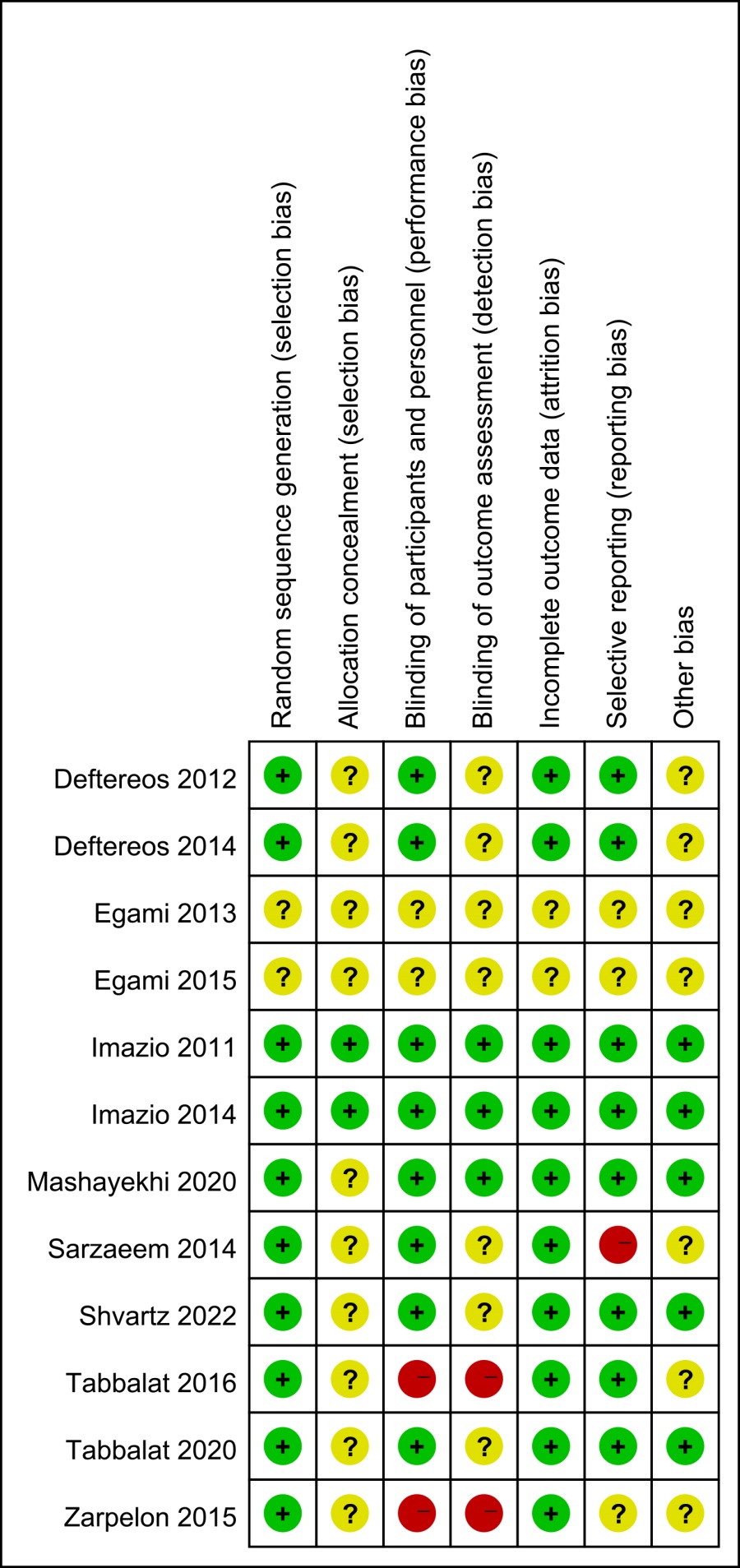


Plus/green suggests low-risk bias, question mark/yellow shows uncertain risk of bias, minus/red indicates a potentially high risk of bias
